# Supplementary material for: The Evolution and Characterization of the RNA Interference Pathways in Lophotrochozoa
Source: Genome Biol Evol. 2024 May 7;16(5):evae098. doi: 10.1093/gbe/evae098 (PMC11114477; doi:10.1093/gbe/evae098)
Supplement: evae098_Supplementary_Data [file evae098_supplementary_data.zip › Supplementary_materials.zip!/selected_miRNAs/NoGe_selected_miRNAs/mirdeep_pdf_files/NMRB01000004.1_Notospermus_geniculatus_strain_Ushimado_scaffold4,_whole_genome_shotgun_sequence_173.pdf]

Provisional ID : NMRB01000004.1_Notospermus_geniculatus_strain_Ushimado_scaffold4,_whole_genome_shotgun_sequence_173
Score total : 769.6
Score for star read(s) : 3.9
Score for read counts : 764.9
Score for mfe : 1.4
Score for randfold : 0
Score for cons. seed : -0.6
Total read count : 1512
Mature read count : 1506
Loop read count : 0
Star read count : 6

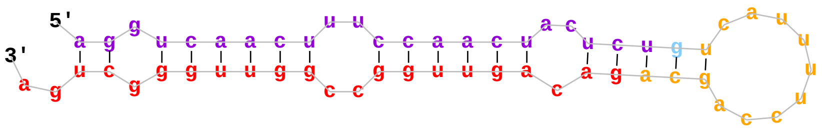

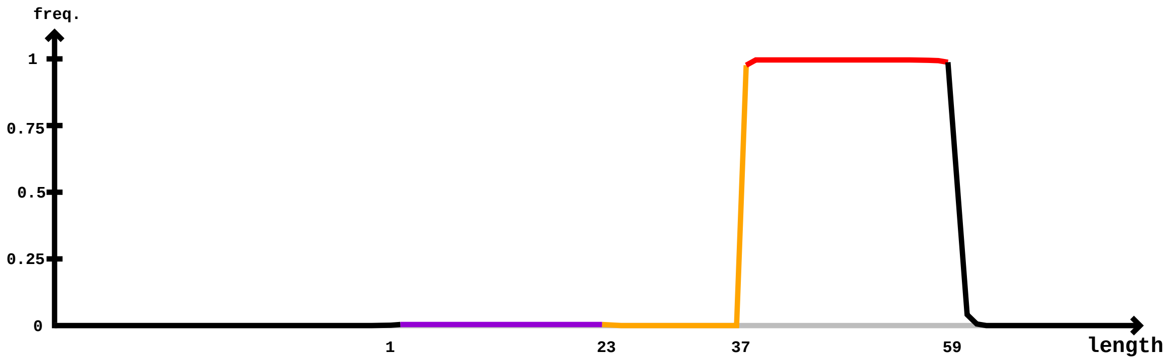

Star **Mature**

```
5'- uuugucagggucugagugaaugguuugugacauaaggucaacuuuccaacuacucugucauuuuccagcagacaguuggccgguugggcugauguuuacagaucagugcau   -3'  obs
    uuugucagggucugagugaaugguuugugacauaaggucaacuuuccaacuacucugucauuuuccagcagacaguuggccgguugggcugauguuuacagaucagugcau        exp
    ....((((...))))(((..(((((((((((((.((.((((((..((((((..(((((.........))))).))))))..)))))).)).))).)))))))))).)))..    reads   mm   sample
    ......................................................................Uacaguuggccgguugggcuga...................    1       1    Fm2
    ......................................................................gacaguuggccgguugggcugG...................    10      1    Fm2
    ......................................................................gacaguuggcGgguugggcuga...................    1       1    Fm2
    ......................................................................gacaguuggccgguuggUcuga...................    2       1    Fm2
    ......................................................................gacaguuggccgguuggAcuga...................    1       1    Fm2
    ......................................................................gacaguuggccgguugAgcuga...................    1       1    Fm2
    ......................................................................gacaguuggccgguugggcuga...................    242     0    Fm2
    ......................................................................gacaguuggccgguugggcugau..................    11      0    Fm2
    ......................................................................gacaguuggccgguugggcugaA..................    25      1    Fm2
    ......................................................................gacaguuggccgguugggcugauU.................    15      1    Fm2
    ......................................................................gacaguuggccgguugggcugauA.................    6       1    Fm2
    .......................................................................acaguuggccgguugggcuga...................    5       0    Fm2
    .......................................................................acaguuggccgguugggcugau..................    3       0    Fm2

    ......................................................................gacaguuggccgguugggcuga...................    203     0    Fm1
    ......................................................................gacaguuggccgguuggAcuga...................    3       1    Fm1
    ......................................................................gacaguuggccgguugggcugU...................    2       1    Fm1
    ......................................................................gacGguuggccgguugggcuga...................    1       1    Fm1
    ......................................................................gacaguuggUcgguugggcuga...................    1       1    Fm1
    ......................................................................gaAaguuggccgguugggcuga...................    1       1    Fm1
    ......................................................................gacaAuuggccgguugggcuga...................    1       1    Fm1
    ......................................................................gacaguuggccgguugggcugG...................    4       1    Fm1
    ......................................................................gacaguuggccgguugggcuAa...................    1       1    Fm1
    ......................................................................gacaguuggccgguugCgcuga...................    1       1    Fm1
    ......................................................................gacaguuggccgguuggCcuga...................    1       1    Fm1
    ......................................................................gacaguuggccgguugggcugau..................    23      0    Fm1
    ......................................................................gacaguuggccgguugggcugaA..................    5       1    Fm1
    ......................................................................gacaguuggccgguugggcugauU.................    8       1    Fm1
    ......................................................................gacaguuggccgguugggcugauA.................    4       1    Fm1
    .......................................................................acaguuggccgguugggcuga...................    2       0    Fm1
    .......................................................................acaguuggccgguugggcugaA..................    3       1    Fm1

    ......................................................................gacaguuggccgguugggcu.....................    2       0    Fm3
```

```
                                 Star                              Mature
uuugucagggucugagugaaugguuugugacauaaggucaacuuuccaacuacucugucauuuuccagcagacaguuggccgguugggcugauguuuacagaucagugcau
......................................................................gacaguuggccgguuggAcug....................    1     1    Fm3
......................................................................gacaguuggcAgguugggcuga...................    1     1    Fm3
......................................................................gacaguuggGcgguugggcuga...................    1     1    Fm3
......................................................................gacaguuggccgguuUggcuga...................    2     1    Fm3
......................................................................gacaguuggccgguuggUcuga...................    1     1    Fm3
......................................................................gacaguuggccggAugggcuga...................    1     1    Fm3
......................................................................gacaguuggccgguugggAuga...................    1     1    Fm3
......................................................................gacaguuggccgguugggcugU...................    1     1    Fm3
......................................................................Uacaguuggccgguugggcuga...................    1     1    Fm3
......................................................................gCcaguuggccgguugggcuga...................    1     1    Fm3
......................................................................gacaguuggcGgguugggcuga...................    2     1    Fm3
......................................................................gacaguuggccgguugAgcuga...................    4     1    Fm3
......................................................................Nacaguuggccgguugggcuga...................    1     1    Fm3
......................................................................gacaguuggccgguugCgcuga...................    3     1    Fm3
......................................................................gacaguuggccgguuggCcuga...................    3     1    Fm3
......................................................................gacagAuggccgguugggcuga...................    1     1    Fm3
......................................................................gacaguuggccgguuggAcuga...................    3     1    Fm3
......................................................................gacaguuggccgguugggcuga...................    524   0    Fm3
......................................................................gacaguuggccgguugggcugau..................    5     0    Fm3
......................................................................gacaguuggccgguugggcugaA..................    16    1    Fm3
......................................................................gacaguuggccgguugggcugUu..................    1     1    Fm3
......................................................................gacaguuggccgguugggcugauA.................    4     1    Fm3
.......................................................................acaguuggccgguugggcuga...................    10    0    Fm3
.......................................................................acaguuggccgguugggGuga...................    1     1    Fm3
.......................................................................acaguuggccgguugggcugau..................    1     0    Fm3

......................................................................gacaguuggccgguugggcug....................    5     0    Mm1
......................................................................gacaguuggccgguugggGug....................    1     1    Mm1
......................................................................gacaguuggccgguugUgcuga...................    1     1    Mm1
......................................................................gacaguuggccgguuggUcuga...................    1     1    Mm1
......................................................................gacaguuggccgguuggAcuga...................    1     1    Mm1
......................................................................gacaguuggccgguugCgcuga...................    1     1    Mm1
......................................................................gacaguuggccgguugggcuga...................    85    0    Mm1
......................................................................gacaguuggccgguugggcugU...................    2     1    Mm1
......................................................................gacaguuggccgguugggcugaG..................    2     1    Mm1
......................................................................gacaguuggccgguugggcugau..................    2     0    Mm1
......................................................................gacaguuggccgguugggcugaA..................    14    1    Mm1
......................................................................gacaguuggccgguugggcugauU.................    6     1    Mm1
......................................................................gacaguuggccgguugggcugaAgu................    9     1    Mm1

................................uaaggucaacuuuccaacuacucu.......................................................    1     0    Mm2
.................................aaggucaacuuuccaacuacucu.......................................................    1     0    Mm2
..................................aggucaacuuuccaacuacucu.......................................................    4     0    Mm2
......................................................................gacaguuggccgguuggg.......................    1     0    Mm2
......................................................................gacaguuggccgguugggc......................    1     0    Mm2
......................................................................gacaguuggccgguugggcug....................    2     0    Mm2
......................................................................gacaguuggccgguugggcuga...................    107   0    Mm2
......................................................................gacaguuggccgguugggcugG...................    4     1    Mm2
......................................................................gacaguuggcAgguugggcuga...................    1     1    Mm2
......................................................................gacaguuggccgguugggcugU...................    2     1    Mm2
......................................................................gacaguuggccgguugggcugaA..................    10    1    Mm2
......................................................................gacaguuggccgguugggcugau..................    6     0    Mm2
......................................................................gacaguuggccgguugggcugUu..................    1     1    Mm2
......................................................................gacaguuggccgguugggcugaG..................    1     1    Mm2
......................................................................gacaguuggccgguugggcugauA.................    4     1    Mm2
......................................................................gacaguuggccgguugggcugauU.................    5     1    Mm2
.......................................................................acaguuggccgguugggcuga...................    4     0    Mm2
.......................................................................acaguuggccgguugggcugau..................    1     0    Mm2

......................................................................gacaguuggccgguugggcuga...................    34    0    Mm3
......................................................................gacaguuggccgguugggcugG...................    1     1    Mm3
......................................................................gacaguuggccgguugggcugU...................    3     1    Mm3
......................................................................gacaguuggccgguugggcugau..................    6     0    Mm3
......................................................................gacaguuggccgguugggcugaA..................    2     1    Mm3
......................................................................gacaguuggccgguugggcugauU.................    1     1    Mm3
```
